# Supplementary material for: Causal interplay between lactose intolerance and gut microbiota: a combined bidirectional Mendelian randomization and in vivo validation study
Source: Front Nutr. 2026 Jun 1;13:1803337. doi: 10.3389/fnut.2026.1803337 (PMC13265576; doi:10.3389/fnut.2026.1803337)
Supplement: Supplementary file 1 [file Data_Sheet_1.zip › supplementary materials/Forward/leave one out cross validation/ebi-a-GCST90027546.finngen_R12_E4_LACTONAS.pdf]

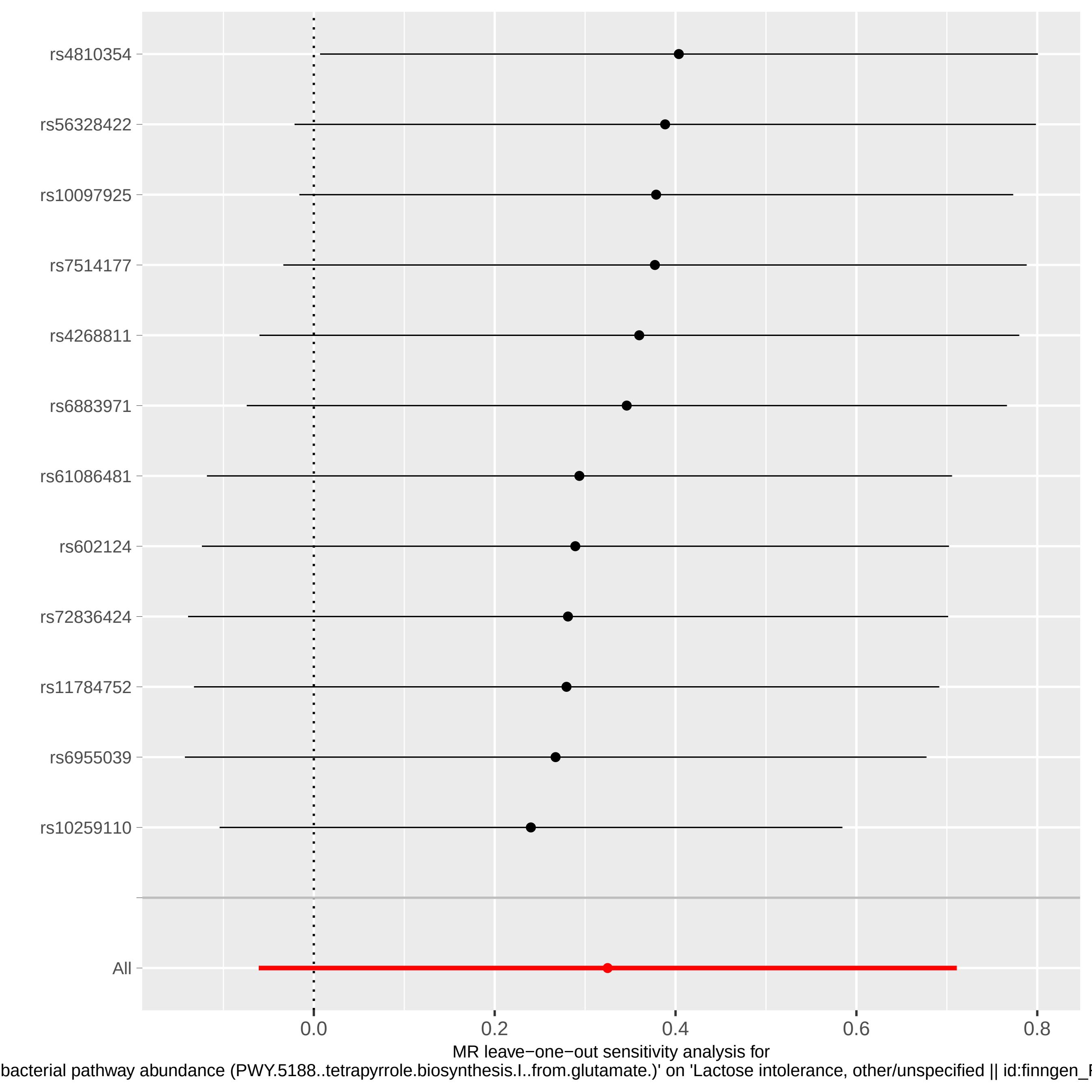
rs4810354
rs56328422
rs10097925
rs7514177
rs4268811
rs6883971
rs61086481
rs602124
rs72836424
rs11784752
rs6955039
rs10259110
All
0.0
0.2
0.4
0.6
0.8
MR leave−one−out sensitivity analysis for
bacterial pathway abundance (PWY.5188..tetrapyrrole.biosynthesis.I..from.glutamate.)' on 'Lactose intolerance, other/unspecified || id:finngen_R
